# Complex refractive index spectra of whole blood and aqueous solutions of anticoagulants, analgesics and buffers in the mid-infrared

**DOI:** 10.1038/s41598-017-07842-0

**Published:** 2017-08-04

**Authors:** David J. Rowe, David Smith, James S. Wilkinson

**Affiliations:** 1 Optoelectronics Research Centre, University of Southampton, Highfield, Southampton SO17 1BJ UK; 2 Faculty of Medicine, University of Southampton, Highfield, Southampton SO17 1BJ UK

## Abstract

Mid-infrared (MIR) spectroscopy is a powerful tool for characterising the vibrations of molecular bonds and is therefore ideal for label-free detection of chemical species. Recent research into thin-film deposition and etching techniques for mid-infrared materials shows potential for realising miniaturised bedside biosensors for clinical diagnostics exploiting MIR spectroscopy, to replace laboratory based-techniques. However, lack of refractive index information for commonly encountered biological media and analytes hampers optimisation of biosensor performance for maximum sensitivity, especially for devices exploiting evanescent spectroscopy. Here we present refractive index data for human whole blood and several aqueous solutions of general interest to the clinical community: anticoagulants, analgesics and buffers. The refractive indices are generally dominated by the water content of each sample and the whole blood spectra exhibit additional strong features due to protein content. Furthermore, we present a generalised method for extracting complex refractive indices of aqueous solutions in the mid-infrared region using conventional attenuated total reflection Fourier transform spectroscopy (ATR-FTIR) without the need for collimated or polarised incident light, as is required for existing methods.

## Introduction

The mid-infrared (MIR) spectral region covering wavelengths from 2 µm to 15 µm is dominated by absorptions corresponding to molecular vibrations, making it suitable for label-free biosensing through direct identification of the molecular “fingerprint”. It is especially suitable for identifying pharmaceutical compounds, offering greater specificity than for macromolecules such as proteins, because the molecules are significantly smaller. This means there are typically both fewer bonds and fewer types of bonds present, resulting in a better defined distribution of absorptions and simpler data analysis, leading to reliable identification or quantification of the chemical species in a given sample.

Infrared spectroscopy has found widespread use as a laboratory technique for compositional analysis over recent decades^1^, especially in the form of Fourier transform infrared (FTIR) spectroscopy. The attenuated total reflection configuration^2^ (ATR-FTIR) is particularly well suited to analysing highly absorbing samples by reducing the path length of interaction between the incident beam and the sample from the submillimetre to the micron scale. This is achieved by total internal reflection of the incident beam within a crystal where the sample is placed in contact with the crystal so that it interacts with the evanescent field. The ATR approach also allows straightforward analysis of the sample matrix in turbid media such as whole blood, as the evanescent field is minimally affected by scatterers such as erythrocytes^3^.

Point-of-care diagnostics, where sample preparation and logistical aspects of biomedical analyses are minimised and analytical devices are brought to the patient, have the potential to revolutionise healthcare. While ATR spectroscopy in the mid-infrared has the potential to achieve this, moving specimen characterisation from the laboratory to the bedside would benefit from low-cost, miniaturised, mass-producible technology allowing handheld diagnostic devices. Economies of scale could potentially yield disposable point-of-care sensors that, unlike benchtop instruments, would not rely on user alignment. This could be achieved for ATR-FTIR spectroscopy using an evanescent waveguide sensor, which is effectively a compact and ultrasensitive ATR element^4^. Techniques for fabricating such mid-infrared devices have been developed by our group^5^ and by others^6–10^, but these devices have not yet been used for characterising the composition of human-derived materials such as blood.

Optimal design of waveguides or ATR elements for MIR spectroscopy requires accurate knowledge of the refractive index of the sample medium, as the evanescent absorption is strongly dependent upon index, due to variation in the evanescent penetration depth. Furthermore, the refractive index excursions which occur near absorption lines distort the measured spectra, so that knowledge of both the real and imaginary (absorptive) parts of the refractive index of samples containing absorbing species is necessary for accurate interpretation of spectra. Thus, *a priori* knowledge of the optical properties of (i) common sample matrices and (ii) samples containing clinically important analytes is needed in order to model the behaviour of mid-infrared evanescent biosensors and optimise the sensitivity and limit of detection for biomedical applications.

Blood composition is critically important throughout medicine, so its refractive index is of widespread significance to the design of mid-infrared biosensors. Refractive index spectrum measurements on whole blood are of particular interest because clinical measurements are ideally carried out in whole blood in order to minimise sample preparation, critically saving time and reducing cost. The MIR absorbance of blood has previously been reported in the wavelength range 2–10 μm^11^ using a single reflection ATR element but there is insufficient information to calculate either component of the complex refractive index.

An exemplar clinical application is the analysis of analgesics in whole blood^12^. Two drugs commonly self-administered in overdose are paracetamol and aspirin^13, 14^, each of which requires a different treatment^15, 16^ in a short timescale. The refractive index spectra of these materials is of interest for designing an optical device to identify the drugs from a blood sample and to quantify their concentrations. Both paracetamol and aspirin have been thoroughly modelled and characterised in the mid-infrared region but not in aqueous solution, as will ultimately be required for point-of-care blood testing, and not in terms of complex refractive index^17–24^.

In practice, the majority of prototype point-of-care sensors will employ *ex vivo* testing rather than *in vivo* testing, necessitating the use of anticoagulants because clotting factors readily cause whole blood to coagulate *ex vivo*, complicating sample handling. To date there are no reported examples of the mid-infrared properties of anticoagulants, and the effect of these on sample refractive index is important for the purposes of interpreting sample spectra in the presence of anticoagulants.

Phosphate-buffered saline and carbonate-bicarbonate are aqueous buffer solutions that are widely used in clinical and analytical settings for biochemical measurements. We anticipate that precise knowledge of the complex refractive index spectra of buffer solutions will become significant as mid-infrared technologies mature and become used for applications such as characterising proteins which have been extracted into buffer and adsorbed onto a waveguide surface.

Therefore we present (i) reference data for the mid-infrared complex refractive index of human whole blood and aqueous solutions of anticoagulants, analgesics and buffers of general interest to the clinical community and which may be used to aid the design of any MIR device for biodiagnostics and (ii) a generalised method for extracting such complex refractive indices of aqueous solutions in the mid-infrared region using conventional ATR-FTIR without the need for collimated or polarised incident light as is required for existing methods. ATR absorption spectra are obtained, calibrated by reference to known water spectra, the real part of index is deduced using the Kramers-Kronig (KK) transform, and the full complex refractive index are generated and can be found at DOI 10.5258/SOTON/405161. We also present the change in complex refractive index for the aqueous solutions relative to water to illustrate how this method can be implemented for sensing applications and allow design of optimised sensing devices. The reference data presented here will enable the optimised design of specific ultrasensitive biodiagnostic modalities, such as waveguide evanescent spectroscopy^3–10^ or resonant antennas^25–27^, for detection of low concentrations of solutes at the point of care.

## Results

### ATR-FTIR absorbance spectra

**Figure 1:**
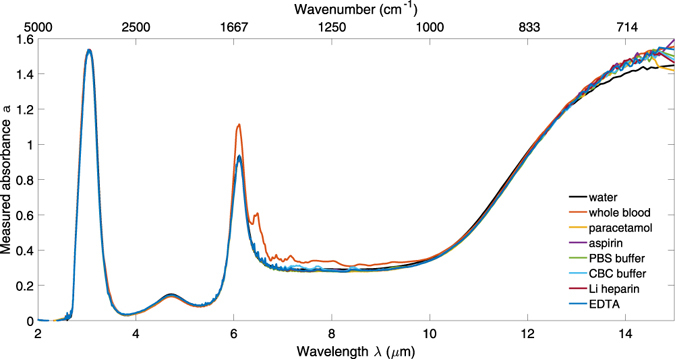
Absorbance spectra of DI water, whole blood and aqueous solutions of paracetamol, aspirin, heparin, EDTA, PBS and CBC.

Figure 1 shows the absorbance spectra for all measured samples, referenced to a nitrogen atmosphere. Clearly the absorption due to water dominates the absorption of all other solutes. The obvious additional features in the whole blood spectrum correspond to those observed previously^11^ and can be attributed to amide bonds, which is to be expected given the high protein content of blood.

### Calculation of the imaginary part of refractive index

In conventional transmission spectroscopy, the imaginary component of refractive index (k) is calculated from MIR absorption spectra using the Beer-Lambert law:

1 $$k(\lambda )=\frac{\lambda a}{4\pi d\,{\mathrm{log}}_{10}e}$$

with wavelength *λ*, path length *d* and absorbance *a* = −log_10_(*I/I*_*0*_ *)* where *I* and *I*_*0*_ are the sample and reference intensities. The path length of the incident beam through the sample is readily known for transmission measurements, for instance in a cuvette, but is not directly observable for an evanescent technique such as ATR-FTIR spectroscopy. Instead it is possible to define an effective penetration depth *d*_*ep*_ equivalent to the thickness of material that would give the same absorption in transmission. This has been derived from a model of evanescent field for a single reflection at the crystal/sample interface^2^, and is wavelength-dependent due to dispersion. In the case of multiple reflections within the ATR crystal to increase the light-sample interaction, *d*_*ep*_ can be multiplied by the integer number of reflections *N*, so that *d* = *Nd*_*ep*_.

This model is strongly dependent on the angle of internal reflection of the incident beam, which should therefore be collimated so the incident angle is constant within a high tolerance. However, the majority of FTIR spectrometers, including the one used here, do not have a collimated beam in the sample chamber since their primary configuration is for transmission measurements. This means the effective penetration model cannot be used directly to calculate *k*.

The depth of penetration is a wavelength-dependent function of the refractive index of the ATR crystal (here, ZnSe), as well as sample index, incident angle and polarisation. For a collimated beam, knowledge of these quantities allows an analytical model for penetration depth, which for an unpolarised beam can be assumed to take the average of the TE and TM polarisations. Whilst the properties of ZnSe are well known, it is not helpful to separate this quantity out in this case because of the unknown range of incident angles in an uncollimated beam which means that *d* is unknown and prevents an analytical solution to equation (1).

In the absence of a well-defined value of *d* we propose to use water, which has known complex index^28^, as a standard to determine an effective penetration depth *d*_*eff*_. *d*_*eff*_ can be calculated by rearranging equation (1) for *d* and using the absorbance of water from our ATR measurement and the imaginary part of index *k* from Hale and Querry^28^ since all other variables and constants are known. Unlike the single angle model discussed above, this method incorporates the contributions of all angles of incidence at each wavelength. This allows calculation of *k* from ATR absorption data for aqueous solutions where the deviation in refractive index from that of water is small, given that the optical behaviour can be expected to be dominated by water because it is so highly absorbing. The empirical estimation of penetration depth therefore accounts for variations in polarisation, sample and ATR refractive index, and contributions from all angles of incidence with respect to wavelength, without having to known each quantity individually.

**Figure 2:**
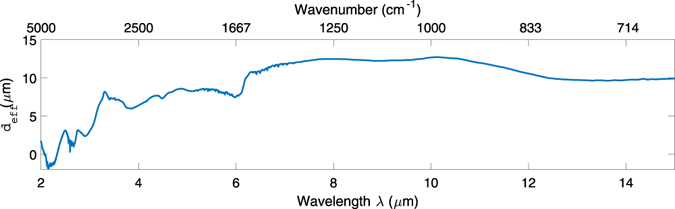
Empirical effective penetration depth *d*_*eff*_ calculated from the measured absorbance and literature *k* values of water. The dashed trace at wavelengths <2.7 μm show the region where *d*_*eff*_ has been calculated from a ratio where both quantities are approximately equal to zero so cannot be relied upon.

The resultant effective penetration depth, *d*_*eff*_, is shown with respect to wavelength in Fig. 2. Note that this is for a ten reflection ATR measurement so *d*_*eff*_ would reduce by a factor of ten for a single reflection. The high noise levels at wavelengths <2.7 μm results from taking the ratio of *k* to *a* in a spectral region where both quantities are approximately equal to zero. Generally there is little biochemical information of interest in this region so it can be ignored. The fluctuations in deduced *d*_*eff*_ at 3 and 6.2 µm reflect the changes in the real part of the index of water at the same wavelengths, altering the index contrast between the sample and the ATR element which strongly affects penetration depth.

**Figure 3:**
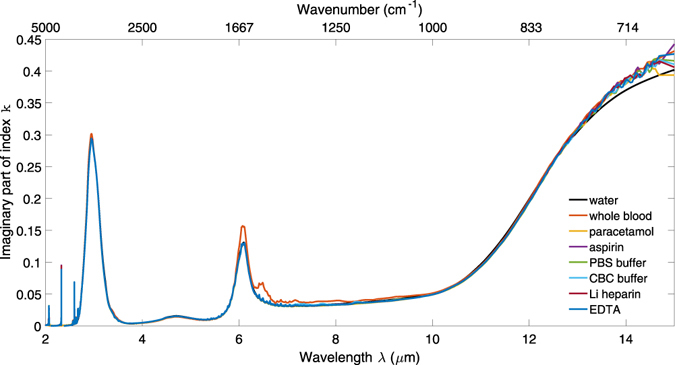
Imaginary part of refractive index spectra k(λ) for whole blood and aqueous solutions of paracetamol, aspirin, heparin, EDTA, PBS and CBC.

The imaginary parts of the refractive index spectra of whole blood and aqueous solutions of anticoagulants, analgesics and buffers can be calculated from equation (1) using their absorbance data and by setting *d = d*_*eff*_. The resultant spectra of *k* are shown in Fig. 3. This procedure allows the accurate determination of the imaginary part of index of aqueous solutions from uncollimated ATR measurements for design and simulation of sensing devices. Clearly the spectra in Fig. 3 are similar, as they are all in aqueous media, and deviations of k from the aqueous background due to the solutes will be presented in detail below.

### Calculation of the real part of refractive index

Modelling of evanescent devices requires knowledge of the real part of the refractive index of the medium as well as the imaginary part. KK transforms can be used to relate the real component of complex data to their imaginary component^29^, so that the real part of refractive index *n* can be calculated from *k* using the algorithms provided by Lucarini *et al*.^30^, for example. The algorithms provide numerical solutions to the general forms.

In theory the entire spectral range of *k* (i.e. from zero to infinite frequency) must be known in order to calculate *n* using KK transforms. Empirically this is not usually possible so extrapolation is required outside the measured frequency range because the calculated *n* is otherwise strongly divergent, even within the measured frequency range. In practice extrapolating to the high frequency range makes little difference because there are no significant spectral features. Extrapolation below the measured frequency range, however, greatly improves the accuracy of the results. Here we extend the imaginary part of the refractive index spectra *k(λ)* of each solution through the region 15 µm–200 µm using published data^28^ because the absorption of water is so strong compared to all other common solvents that it can be assumed to dominate over the entire electromagnetic spectrum. This is supported by a far-infrared comparison of water and blood sera^31^ which found no difference between the two at wavelengths above 14.3 µm.

The result for the real part of index is obtained in terms of susceptibility and must be shifted by a wavelength-independent index offset to obtain *n*. This offset is obtained by comparing the KK result with the known index at one wavelength, usually from a region of low loss where no depolarisation mechanisms are observed. Examples of depolarisation mechanisms are dipolar reorientation in the microwave region, molecular vibrations in the MIR region and electron density distortions in the visible and ultraviolet regions. In response to an applied electric field these mechanisms cause observable features in permittivity and refractive index spectra. For extracting the refractive index of aqueous solutions we choose to obtain the offset from the high frequency region of water near 2 µm where there are no spectral contributions from depolarisation of the analytes in solution.

**Figure 4:**
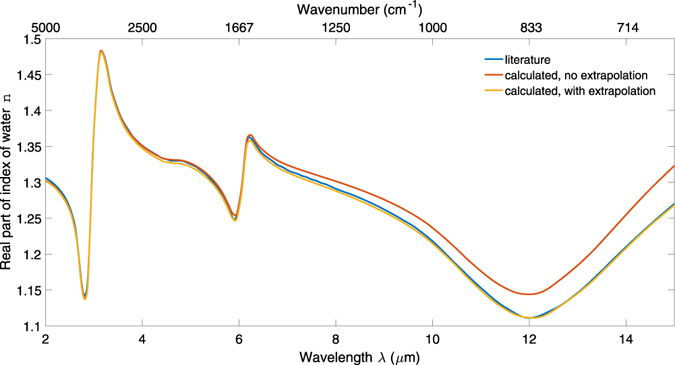
The real part of refractive index of water reported in literature compared with the real index calculated from mid-infrared measurement. The yellow and red traces show the responses with and without low frequency extrapolation of the *k* spectrum used in the calculation.

Figure 4 gives plots of *n* vs *λ* for water from literature^28^ compared with those calculated from the measured *k* following extrapolation, KK transformation and offset as described, and good agreement is achieved. The calculated responses with and without low frequency extrapolation of *k* are both shown, and the response without extrapolated *k* can be seen to be strongly divergent at longer wavelengths.

**Figure 5:**
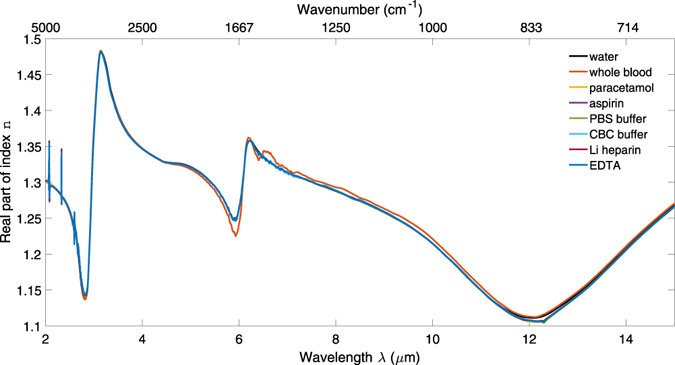
Real part of refractive index spectra n(λ) for whole blood and aqueous solutions of paracetamol, aspirin, heparin, EDTA, PBS and CBC.

This method allows accurate determination of the complex refractive index spectra of aqueous solutions of relatively weakly absorbing solutes, by calibration with water as a simple standard. The real parts of the refractive index spectra of whole blood and aqueous solutions of anticoagulants, analgesics and buffers calculated using the offset and extrapolation described above are shown in Fig. 5.

### Differential complex refractive index of solutions

**Figure 6:**
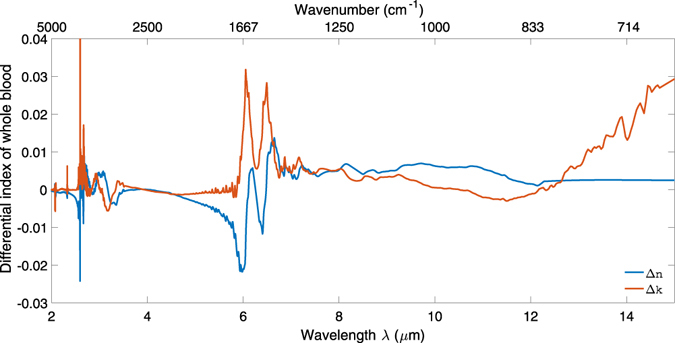
Change in the real and imaginary parts of refractive index for whole blood with respect to water.

For large changes in *n* and *k*, for example between water and whole blood, it is possible to subtract the index spectrum of the solvent from that of a solution or suspension to obtain the differential index spectra of the analyte. The results for both components of complex refractive index of whole blood with respect to water are shown in Fig. 6.

It is common practice to obtain FTIR absorbance spectra by measuring, for example, a solution relative to its pure solvent. This can be a more useful way of collecting data because it removes the background signal from the solvent in order to emphasise spectral features of the analyte. It should be noted that this yields a differential absorbance instead of an absolute absorbance. Here we posit extending the use of differential absorbance measurements to calculating differential refractive indices. This provides an estimate of the refractive index increment due to an analyte whilst excluding the features of a highly absorbing solvent such as water. As with absorbance measurements, this yields differential refractive indices instead of absolute refractive indices. This could be useful in optical designs requiring high sensitivity for detecting low concentration analytes as well as large dynamic range because of a strong background signal. This technique is useful because such information is not currently available.

Incremental changes in refractive index due to dissolved analytes can also be calculated using the KK procedure, to optimise evanescent device designs for specific applications and to allow quantification of analyte concentrations in use. In this instance absorbance is measured with respect to a reference measurement of DI water rather than N_2_-purged atmosphere. Taking the ratio of an aqueous solution to water has the effect of removing the solvent absorption so the resultant response is a differential absorption spectrum due to the analyte only. Refractive index can then be calculated from the data using the method above, differing in that the result is an incremental change in index relative to water rather than an absolute refractive index. This approach minimises variations due to the background medium.

Although the effect of water is removed from the absorbance, the change in the imaginary part of index can be calculated using the same estimation of *d*_*eff*_ because water will still determine the effective penetration depth of the evanescent field from the ATR crystal, as it still present during the measurement and the perturbation in n due to the low concentration analytes is expected to be small. The change in the real part of index can be calculated using the KK transform as above, except that it is unnecessary to offset the data because the background absorption of water has already been corrected for.

Figure 6 shows the change in both components of refractive index for all the aqueous solutions with respect to water. Each column shows a different wavelength region of interest: the first two columns for the analgesics, and the third column for the anticoagulants and buffers. The data were smoothed using a Savitzky-Golay filter with a cubic polynomial and a window of 5 points.

## Discussion

These measurements show that ATR spectroscopy is capable of characterising analytes such as analgesics in aqueous solution, despite strong water absorption. However, to maximise the dynamic range and minimise the limit of detection for such an application using a high-sensitivity evanescent device such as a MIR waveguide, the optical properties of both the background matrix (water, blood, and buffer) and the dissolved analytes must be known. This work demonstrates that each of the materials tested has a complex refractive index spectrum broadly similar to that of water, so that these values can be used for initial device design.

**Figure 7:**
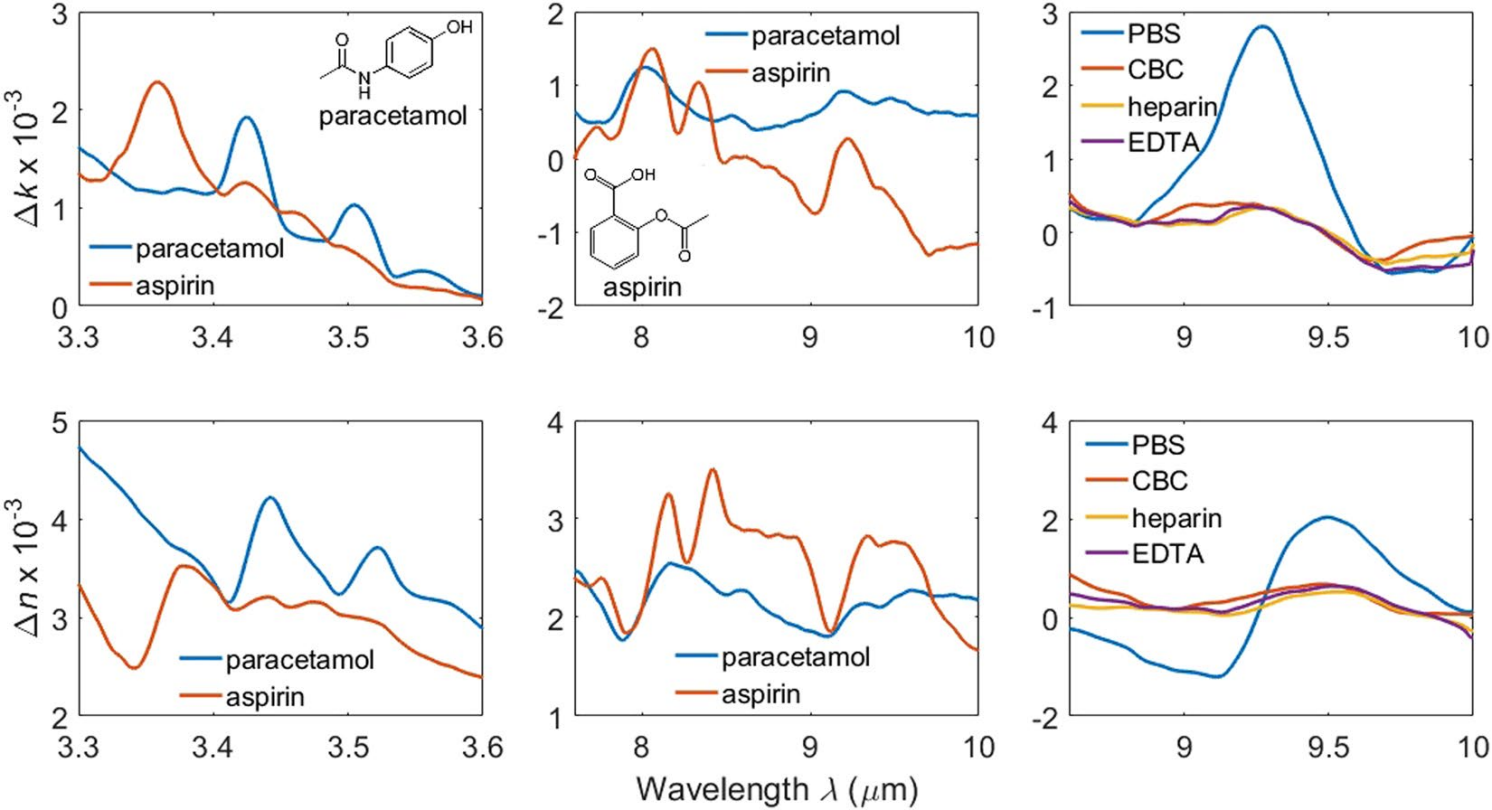
Change in the imaginary (top row) and real (bottom row) parts of refractive index for all the aqueous solutions with respect to water. Each column shows a different wavelength region of interest: the first two columns for the analgesics, and the third column for the anticoagulants and buffers. The molecular structures of paracetamol and aspirin are shown inset.

Water absorbs strongly around λ = 3 μm due to the O-H stretch vibration and at 6.1 μm due to the H-O-H bending vibration. It also absorbs strongly at wavelengths λ > 10 μm due to the L_2_ libration. The differential index spectra of whole blood relative to water, shown in Fig. 6, shows that the most significant additional absorption in *k* due to the amide II absorption at 6.49 μm, with corresponding features in *n*. It also features an amide I absorption at 6.05 μm, which overlaps with the H-O-H absorption of water and causes a larger absorption at this wavelength compared with the other solutions in Figs 1 and 3. If the regions of high background absorption near 3 μm and 6 μm and of potential interference from proteins between 6 μm and 6.5 μm in whole blood are avoided, these results suggest that a sensor with sufficient dynamic range and sensitivity to characterise analytes in aqueous solution will be suitable for whole blood analysis. As an example, the 3.3–3.6 μm and 8–10 μm wavelength regions are suitable for distinguishing between paracetamol and aspirin, as shown in Fig. 7.

The refractive index of whole blood has been extensively characterised and modelled in the visible and near-infrared regions^32–36^, particularly in terms of the scattering behaviour of the different components, and has been comprehensively reviewed^37^. Characterisation with an evanescent technique is much simpler because cellular components, which are the primary cause of scattering in the visible and near-infrared regions, are much larger than the penetration depth of the evanescent field and therefore do not contribute strongly to the measurement, as observed in ref. 3 for evanescent spectroscopy of blood at visible wavelengths. This is true even at MIR wavelengths, as described in ref. 38, where it is observed that the spectra in the MIR are similar for untreated samples and the separated serum, and only differ for lysed samples. This is further evinced here for the MIR by the fact that the whole blood spectrum is so close to that of water, except with additional protein content. This would not be so if blood cells were a major contributor to mid-infrared index as they would tend to reduce the volume-averaged index. Because blood cells do not perturb the MIR evanescent field, they are excluded from the measurement so we have effectively characterised the plasma component of whole blood. During the experiments, no significant adhesion of cellular components to the ATR surface was observed.

The anticoagulant, analgesic and buffer spectra demonstrate that the mid-infrared refractive index of their solutions is dominated by their water content. Using difference spectra with respect to water, it can be seen that the anticoagulants and buffers show very little difference except for a large phosphate absorption for PBS at 9.26 μm, which can be attributed to the large proportion of phosphate salts in the buffer. The lack of spectral differences between the anticoagulants mean it is not important which one is used for the purposes of optical characterisation; it can be assumed to have minimal interference with mid-infrared evanescent spectra of blood. The buffer data are provided for reference. It is anticipated that because of their ubiquity in the life sciences, CBC and particularly PBS will be modelled increasingly frequently as mid-infrared technology matures.

The analgesic difference spectra in Fig. 7 clearly show how difference spectra can be used for identification and quantification of a solute under test. The 3.3–3.6 μm absorptions are characteristic of symmetric and asymmetric C-H stretching vibrations, and the 8–10 μm absorptions are characteristic of aromatic ring flexing vibrations. From the similarities in the structure of aspirin and paracetamol, shown inset in Fig. 7, it is to be expected that they should be identified from differences in the relative magnitude of their absorptions as opposed to having uniquely identifying absorptions. The frequency of the absorption peaks correspond to earlier infrared absorption studies of both analgesics^17–24^.

The analgesic clinical limit of detection for acute overdose is 660 μmol/L^15, 16^, which is approximately thirty times lower than the concentrations presented here. It was not possible to measure meaningful spectra for lower concentration analgesic solutions but the differential change in refractive index is a minimum of ten times greater than its standard deviation throughout the spectra, demonstrating that the signals are statistically significant. The standard deviation for Δn is 2 × 10^−5^ and for Δk is 8 × 10^−5^ throughout the spectra. This shows that the particular conventional ATR-FTIR configuration used here would have too high a limit of detection to characterise analgesic overdose in a clinical setting without further optimisation. However a device specifically optimised to detect concentration changes would be expected to exhibit substantially improved SNR, potentially at the expense of the broadband operation required for general instruments. We estimate that a device exibiting 3 to 10 times higher sensitivity would achieve the required detection limit.

The difference spectra provide information on which spectral regions are diagnostically useful, and also allow estimation of limits of detection in fully optimised waveguide devices where sensitivity to concentration changes in these regions is maximised and instabilities minimised. For *in vivo* characterisation it would be most appropriate to optimise a sensor design in the wavelength region 8–10 μm. This is corroborated by Fig. 6, which shows the differential refractive index spectra of whole blood relative to water and clearly shows that there is little difference between the spectra, for both the real and imaginary parts, in this wavelength region. Waveguide sensors have the potential for internal referencing for absorption spectroscopy and integration of interferometers for direct measurement of refractive index changes down to 10^−8^ ^39^. We are currently in the process of a detailed waveguide device design for this purpose.

Another approach to finding both the real and imaginary parts of refractive index is to use KK transforms to calculate phase from an ATR spectrum and then deduce complex refractive index^40–42^. Bertie and Lan note if the index of the incident (ATR element) medium is not constant with respect to frequency then the KK transforms are approximate because of the variation in incident angle, although this can be corrected to an acceptable accuracy using a wavenumber-dependent correction^37^. However, such a method is not applicable to having a non-collimated beam, which gives a wide range of incident angles that cannot be corrected for so this method cannot be used to calculate index.

While we are targeting the optimisation of evanescent sensors for blood solutes, the method presented here can be generally applied to liquid analytes using ATR systems without collimation or polarisation of the incident beam, for extracting refractive index data for any application. This is simpler than the processes described in the literature heretofore and more representative of a typical ATR-FTIR spectrometer configuration.

## Conclusion

We describe a method for extracting complex refractive index spectra from ATR-FTIR absorption measurements of aqueous solutions without requiring collimation or polarisation of the incident beam. We report the refractive index spectra of clinically-relevant materials to facilitate the optimisation of evanescent sensor design for appropriate dynamic range and sensitivity. We also report the change in complex refractive index spectra to show how this method can be applied to identifying analgesics in solution, and to allow modelling of evanescent sensors for this application. All data presented in this article are openly available at http://dx.doi.org/10.5258/SOTON/405161, allowing direct use in simulations.

## Methods

Acetylsalicylic acid (aspirin, 99% purity), 4-Acetamidophenol (paracetamol, 98% purity), phosphate buffered saline (PBS) tablets and carbonate-bicarbonate tablets (CBC) were sourced from Sigma. Lithium heparin and EDTA anticoagulant Vacutainer tubes were obtained from Becton Dickinson. All chemicals were used as received. The analgesics were weighed and dissolved in DI water at 18.5 mmol/L concentration. The buffer tablets were made up with the specified volume of DI water and HCl was added to obtain the correct pH. The anticoagulants were made up with the specified volume of DI water. The solutions were stored in closed containers and left to equilibrate at 25 °C in a temperature and humidity controlled environment for several hours. DI water was also characterised.

Whole blood samples were collected in accordance with standard clinical research practice. Informed consent was obtained from all participants, but specific ethical approval for the sampling was not required by the Faculty of Medicine Ethics Committee. The samples were labelled anonymously, no volunteer details or identifying information were recorded, and the samples were not stored after characterisation. 10 ml whole blood samples were collected with lithium heparin Vacutainer tubes by venepuncture of three healthy volunteers. The samples were used within two hours of collection and were destroyed immediately after characterisation.

Mid-infrared characterisation was performed with an Agilent Cary 630 FTIR spectrometer and Pike HATR attachments. The ATR element was a 10 reflection, 45 degree ZnSe crystal. Spectra were recorded at 4 cm^−1^ resolution in the wavenumber range 6000–600 cm^−1^ (wavelengths 1.67–16.7 µm) with 32 averaged repeats per measurement. 1 ml of each sample was pipetted into the sample trough, characterised in triplicate and taken relative to the N_2_-purged sample chamber atmosphere. Each blood sample was characterised in triplicate; the mean of these values was used for the calculations. The spectra were baseline corrected, normalised to the O-H stretch vibration at λ = 3 μm, and CO_2_ absorptions removed.

KK transforms derive directly from the principle of causality and describe the relationship between in-phase and out-of-phase response of a system to a sinusoidal perturbation^29, 30^. They are a type of Hilbert transform. The KK transforms are formulated for a complex function χ(*ω*) = *χ*_1_(*ω*) + *χ*_2_(*ω*) of a complex variable *ω*. The two transforms use an integral of one component of *χ*(*ω*) to find the other component for the limits −∞ < *ω* < ∞. In the context of optical spectroscopy this allows the real component of a complex refractive index spectrum to be calculated from the imaginary component, and vice versa. In this article we first calculate the imaginary component of refractive index from absorption spectra, then calculate the real component. The KK transform for calculating the real component from the imaginary component is given in equation (2).

2 $${\chi }_{1}(\omega )=\frac{1}{\pi }P{\int }_{-\infty }^{\infty }\frac{{\chi }_{2}(\omega \text{'})}{\omega \text{'}-\omega }d\omega \text{'}$$

where *P* is the Cauchy principal value.

The weak radiation of an FTIR spectrometer means that non-linear optical effects can be discounted, which simplifies the analysis. Lucarini *et al*. freely provide a Matlab program for numerically computing KK transforms for linear optical materials with the restriction that the data must be evenly spaced in frequency^30^, and this program was employed here.
